# Rare Case of Cocaine-Induced Aortic Aneurysm: A Near Dissection Event

**DOI:** 10.1155/2017/1785410

**Published:** 2017-12-14

**Authors:** Ranju Kunwor, AnnMarie Canelas

**Affiliations:** Department of Internal Medicine, MacNeal Hospital, Berwyn, IL, USA

## Abstract

Cocaine use has been associated with cardiovascular complications such as coronary atherosclerosis, coronary artery spasm, cardiac arrhythmias, acute myocardial infarction, myocarditis, and dilated cardiomyopathies. Aortic dissection is a rare but life-threatening complication of cocaine use. Cocaine and stimulant use can cause aortic aneurysm by increasing the aortic wall stress, and the most feared complications are dissection, rupture, and death. There are no clear guidelines about screening cocaine abusers with CT scan of the chest. We do not know if the number of years of cocaine use or the amount of cocaine use can be associated with higher incidence of aortic aneurysm or dissection. Cocaine-induced aortic aneurysm does not have any specific clinical feature. Common presentation is chest discomfort or chest pain. This common presentation is bewildering enough for clinicians to think of more common causes of chest pain like myocardial infarction and myocarditis. The sudden onset of severe, sharp, stabbing chest or back pain is suggestive of aortic dissection. Here, we present a young otherwise healthy patient with chronic cocaine use presenting with chest pain and found to have significant size aortic aneurysm.

## 1. Introduction

Thoracic aortic aneurysm occurs due to increased aortic wall stress from hypertension and less commonly due to genetic and inflammatory condition or prior cardiac surgery [1]. The most feared complication is dissection [1, 2]. In the general population, aortic dissection occurs in 2.6–3.5 per 100,000 patient years [3]. In a study, 921 cases of acute aortic dissection presenting to IRAD (International Registry for Aortic Dissection) centers from 1996 through 2000 were studied, and only 5 (0.5%) cases were associated with cocaine use (*p* value 0.068). Cocaine seemed to have played a significant role in precipitating aortic dissection among young cohort (age 44.4 ± 3.9 years, *p* value < 0.001) [4]. This IRAD study suggested that cocaine is not likely to be responsible for >1% of aortic dissections.

## 2. Case Presentation

### 2.1. History

A 41-year-old white male with no significant past medical history presented in the emergency department (ED) with chest pain. The patient was having chest discomfort for one day. On the day of presentation, he had severe 10/10 intensity, central chest pain, aggravated with lying down and relieved with sitting up. He had associated dyspnea but denied fever, cough, presyncope, syncope, or palpitations. He denied any similar history in the past. There were no known heart murmurs. There was no history of sudden cardiac death or heart disease in family. He snorted cocaine every two weeks for several years, and the last use was a night before his symptoms started. He admitted to drinking alcohol in weekends but denied tobacco smoking.

### 2.2. Physical Exam

His vital signs in the ED was significant for tachycardia, heart rate 110 beats/minute. Initial BP (blood pressure) in ED was 136/83 mmHg. On exam, he had blowing, decrescendo, grade II/VI diastolic murmur heard best in the third left intercostal space, and grade III/VI holosystolic murmur on the left sternal border. He had crackles at lung base but no pedal edema or jugular venous distension on exam.

### 2.3. Investigation and Management

**Figure 1:**
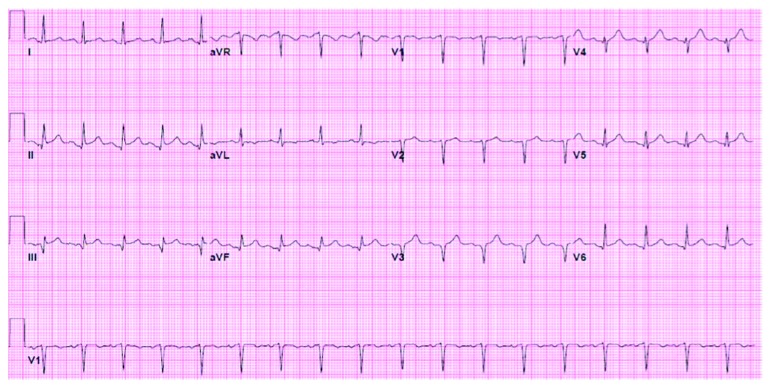
Initial EKG in ED. Rate 108/min. Sinus tachycardia. Anterior, inferior lead ST elevation (QTc 455).

**Figure 2:**
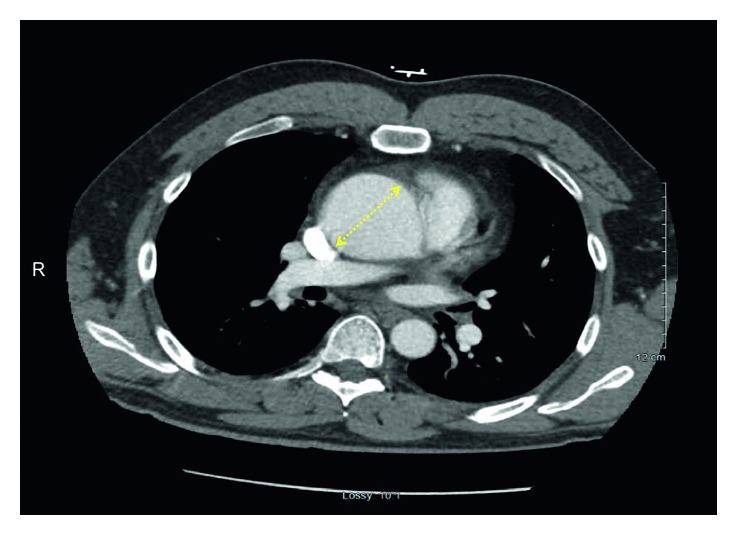
CT chest with contrast showing 6 cm aortic root (yellow dotted line).

**Figure 3:**
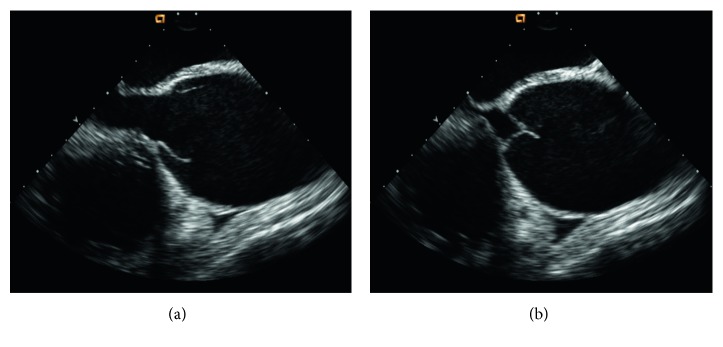
Transesophageal echocardiography (midesophageal, long-axis view) showing massive dilation of ascending aorta. (a) Opening of the aortic valve; (b) closure of the aortic valve.

EKG showed ST elevation suggestive of anterior and inferior infarct (Figure 1). Serial troponins three times were normal. Urine drug screen was positive for cocaine. Chest X-ray showed mild cardiomegaly. The patient was admitted to the telemetry unit with presumptive diagnosis of pericarditis versus cocaine-induced coronary vasospasm. Transthoracic echocardiography (TTE) was performed for the concern of diastolic murmur and pericarditis. TTE showed 6.2 cm aortic root dilatation and severe AR (aortic regurgitation) with possible aortic dissection, EF 50%, and minimal MR (mitral regurgitation) and TR (tricuspid regurgitation). The patient was transferred to the intensive care unit (ICU). The patient's systolic BP increased to the range of 150–160 mmHg. He was started on labetalol for better BP control. CT chest showed 6 cm aneurysm of the aortic root and ascending aorta and aneurysm of the aortic arch 3.6 cm, without dissection (Figure 2). The patient was taken to the operating room (OR) for emergent surgical repair and aortic valve replacement. Transesophageal echocardiography (TEE) was done in the OR. TEE finding was consistent with 6.2 cm aneurysm, moderate to severe AR but no dissection (Figure 3). The aortic valve was trileaflet. Emergency repair of ascending aortic aneurysm using a Hemashield graft, and aortic valve replacement using a mechanical prosthesis, with repair of the sinus of Valsalva aneurysm was performed.

Biopsy results/pathology showed the aortic valve with degenerative changes but no cystic medial degeneration. Aortic tissue comprised large vessel walls, with hemorrhage and foci of acute and chronic inflammation. This is a nonspecific finding and can be seen in hypertensive aortic aneurysm and/or dissection. Cocaine can cause profound sympathetic stimulation and hypertension that presumably causes sheer stress on the aorta's intima that a small “Nick” or tear occurs which explains the hemorrhage in the pathology.

Blood cultures were negative. ANA panel was done for the concern of vasculitis and was negative. Marfan's gene was not tested given the pathology finding, absent Marfanoid clinical features, and negative family history. Postsurgical TTE shows prosthetic aortic valve, with no evidence of aortic regurgitation. EF 50%. Minimal MR. Mild TR. Postoperatively, he was started on heparin bridge to warfarin for anticoagulation. Because the patient had borderline low EF of 50% pre- and postoperatively, the cardiologist recommended starting the patient on metoprolol, furosemide, and lisinopril. The patient was discharged to home on postoperative day number 5.

## 3. Discussion

This case illustrates that cocaine can be a predisposing factor for aortic aneurysm and/or dissection in otherwise healthy, young, and not genetically predisposed person [5]. Our patient had trileaflet aortic valve, negative family history, and no medial degeneration (previously designated cystic medial necrosis) in the pathology, thus making the congenital process less likely. There was no trauma or hypertension history. We did not think of doing CT at the initial presentation in this patient. ACC/AHA guideline does mention cocaine as a risk factor for thoracic aneurysm, but it does not have screening guidelines in cocaine abusers [6]. The question is, is it worth doing CT chest in every cocaine abuser presenting with chest pain? No clear answer to this question exists, but there is a high suspicion for etiology, considering CT in appropriate patients is reasonable. Although cocaine is a rare cause of this life-threatening and rare diagnosis [3], astute suspicion will help prevent a fatal consequence [1, 7].
